# Quantum optical tomography based on time-resolved and mode-selective single-photon detection by femtosecond up-conversion

**DOI:** 10.1038/s41598-023-48270-7

**Published:** 2023-11-29

**Authors:** Naoto Namekata, Nobuaki Kobayashi, Kenya Nomura, Tokuei Sako, Norio Takata, Shuichiro Inoue

**Affiliations:** 1 Institute of Quantum Science, Nihon University, 1-8-14 Kanda-Surugadai, Chiyoda-Ku, Tokyo, 101-8308 Japan; 2 Department of Precision Machinery Engineering, College of Science and Technology, Nihon University, 7-24-1 Narashinodai, Funabashi, Chiba 274-8501 Japan; 3 Laboratory of Physics, College of Science and Technology, Nihon University, 7-24-1 Narashinodai, Funabashi, Chiba 274-8501 Japan; 4 Division of Brain Science, Institute for Advanced Medical Research, Keio University School of Medicine, 35 Shinanomachi, Shinjyuku, Tokyo, 160-8582 Japan

**Keywords:** Biological techniques, Engineering, Optics and photonics

## Abstract

We developed an optical time-of-flight measurement system using a time-resolved and mode-selective up-conversion single-photon detector for acquiring tomographic images of a mouse brain. The probe and pump pulses were spectrally carved from a 100-femtosecond mode-locked fiber laser at 1556 nm using 4f systems, so that their center wavelengths were situated at either side of the phase matching band separated by 30 nm. We demonstrated a sensitivity of 111 dB which is comparable to that of shot-noise-limited optical coherence tomography and an axial resolution of 57 μm (a refractive index of 1.37) with 380 femtosecond probe and pump pulses whose average powers were 1.5 mW and 30 μW, respectively. The proposed technique will open a new way of non-contact and non-invasive three-dimensional structural imaging of biological specimens with ultraweak optical irradiation.

## Introduction

Optical coherence tomography (OCT) is a well-established non-contact and non-invasive method for three-dimensional structural imaging of complex specimens^1^. This technique is actively used for various biomedical applications, such as medical diagnosis. However, the relatively shallow penetration depth, i.e. the observable limit in depth, for biomedical specimens like a mouse brain is considered a serious limitation for many biological and medical applications.

The image formation of OCT relies on the interferometric measurement of backscattered photons from various depths and scattering sites in the specimen. These photons can be classified into singly reflected “ballistic” photons and multiply scattered photons^2,3^. Singly reflected ballistic photons contribute to the meaningful OCT signal, while multiply scattered photons essentially add to the background noise.

The penetration depth of OCT is fundamentally limited by the attenuation of ballistic light propagation via scattering and absorption. The water absorption has local minima in near infrared (NIR) window I (700 – 900 nm) and II (1100 – 1350 nm), therefore the center wavelength is selected in the windows for specimens that contain relatively much water, e.g. a retina. On the other hand, since scattering losses are inversely proportional to the wavelength of light relative to the size of the scattering features, the penetration depth of OCT would benefit from employing a longer center wavelength beyond NIR Window I and II. However, the water absorption loss increases significantly at around 1500 nm and beyond 1850 nm^4,5^. Therefore, the spectral window of 1550 – 1800 nm referred to as NIR Window III or “golden window” would provide optimal performance for enhancing image contrast at larger penetration depths. In fact, OCT in the 1700-nm spectral band has been developed in the last few years^6–8^.

Besides the scattering and absorption losses, the phenomenon of multiple scattering also makes it difficult to achieve meaningful structural information at deeper penetration depths^9^. The fraction of multiply scattered photons that contributes to the OCT image increases with the depth in the specimen, resulting in reduced signal localization and decreased image contrast at larger depths^9–11^. Moreover, OCT suffers from speckle noise that effectively causes significant degradation in spatial resolution^12^. Speckle noise is inherent to all coherent imaging methodologies and arises from the interference of light scattered from multiple points within a turbid specimen, such as biological tissue^13^.

Optical time-of-flight measurements employed in light detection and ranging (LIDAR) systems provide an alternative way to acquire three-dimensional structural images. The axial resolution is mainly limited by the timing jitter (or temporal resolution) of photodetectors. To achieve the axial resolution comparable to OCT, the timing jitter must be less than 100 fs which correspond to the axial resolution of 15 μm in air. The ultrashort timing jitter can be accomplished by up-conversion detectors which utilize sum-frequency generation (SFG) in a χ^2^ nonlinear crystal where pump pulses typically translate the wavelength of signal photons from IR to visible or near IR^14,15^. Note that the pump pulses create a photodetection window independent of detector’s timing jitter and its associated electronics. The temporal resolution of the up-conversion detector depends on the pump pulse width as well as the timing jitter between the signal and the pump pulses caused by the group velocity difference in the nonlinear crystal. Noncolinear broadband up-conversion in periodically poled MgO-doped stoichiometric lithium tantalate with an ultrafast pump and the detection by Si single-photon avalanche diodes enabled efficient detection of ~ 1550 nm IR photons with a temporal resolution of ~ 150 fs^16^.

The optical time-of-flight measurements with the femtosecond time-resolved up-conversion detector in NIR Window III would reduce the scattering and absorption losses in the biological tissue and enhance the penetration depth with the axial resolution of several micrometers. Since the imaging based on the optical time-of-flight measurements is not a coherent imaging, acquired images are less affected by speckle noise. To enhance image contrast at larger penetration depths, multiply scattered photons must be removed. Although they overlap in both spectral and time domains with the singly reflected signal photons, they might have different polarizations and spatiotemporal modes due to multiple scattering. Therefore, a mode-selective photodetection would effectively remove the multiply scattered noise photons.

The mode-selective photodetection can be achieved through the so-called “quantum pulse gate” which was originally developed for quantum information processing using high-dimensional photons^17,18^. The quantum pulse gate is based on the SFG where the group velocity of the pump wave is engineered to match that of either the signal or the sum frequency wave while making it very different from the other^19–21^. However, only a few nonlinear materials can be operated in the “single sideband velocity matched” regime. Then the mode-selective up-conversion by modulating pump pulses instead was proposed by Huang and Kumar to avoid the need for dispersion tailoring of the nonlinear medium^22^. Here, the temporal width $\Delta {T}_{p}$ of the pump pulse must be comparable to or shorter than the reciprocal of the phase-matching bandwidth $\Delta {B}$ of the nonlinear medium, i.e., $\Delta {T}_{p}\Delta {B}\le 1$. Under the condition, only signal photons in a single spatiotemporal mode, whose profile can be tailored by shaping the pump pulses with optical arbitrary waveform generation, are efficiently converted. Undesirable photons overlapping temporally and spectrally but in orthogonal time–frequency modes as well as broadband noise are converted with much less efficiency^23,24^. The mode selectivity was demonstrated by achieving detection signal to noise more than 40 dB over a linear-optical filtering and detection system and beat the theoretical limit of an ideal matched filter by 11 dB^25,26^. Furthermore, the three-dimensional active imaging through opaque and multi-scattering media was demonstrated, where the background is orders of magnitude stronger than the signal^27,28^.

To achieve high conversion efficiency, the pump and signal waves are routinely prepared in narrowband spectral profiles well contained in the SFG phase matching bandwidth. The time-resolved up-conversion detection in reference^16^ employed a 1-mm-long nonlinear waveguide for ensuring the broader phase matching bandwidth than the spectral profiles of the femtosecond pump and signal pulses. However, by employing a longer nonlinear waveguide with a narrower phase matching bandwidth, the efficient mode-selective condition $\Delta {T}_{p}\Delta {B}\ll 1$ can be satisfied, i.e., both a femtosecond temporal resolution and a single temporal mode selection are attainable.

In this work, we developed an optical tomography system based on the time-resolved and mode-selective single-photon detection by femtosecond up-conversion and demonstrated the feasibility of acquiring a tomographic image of strongly light-scattering materials.

## Quantum optical tomography system

### Experimental setup

**Figure 1:**
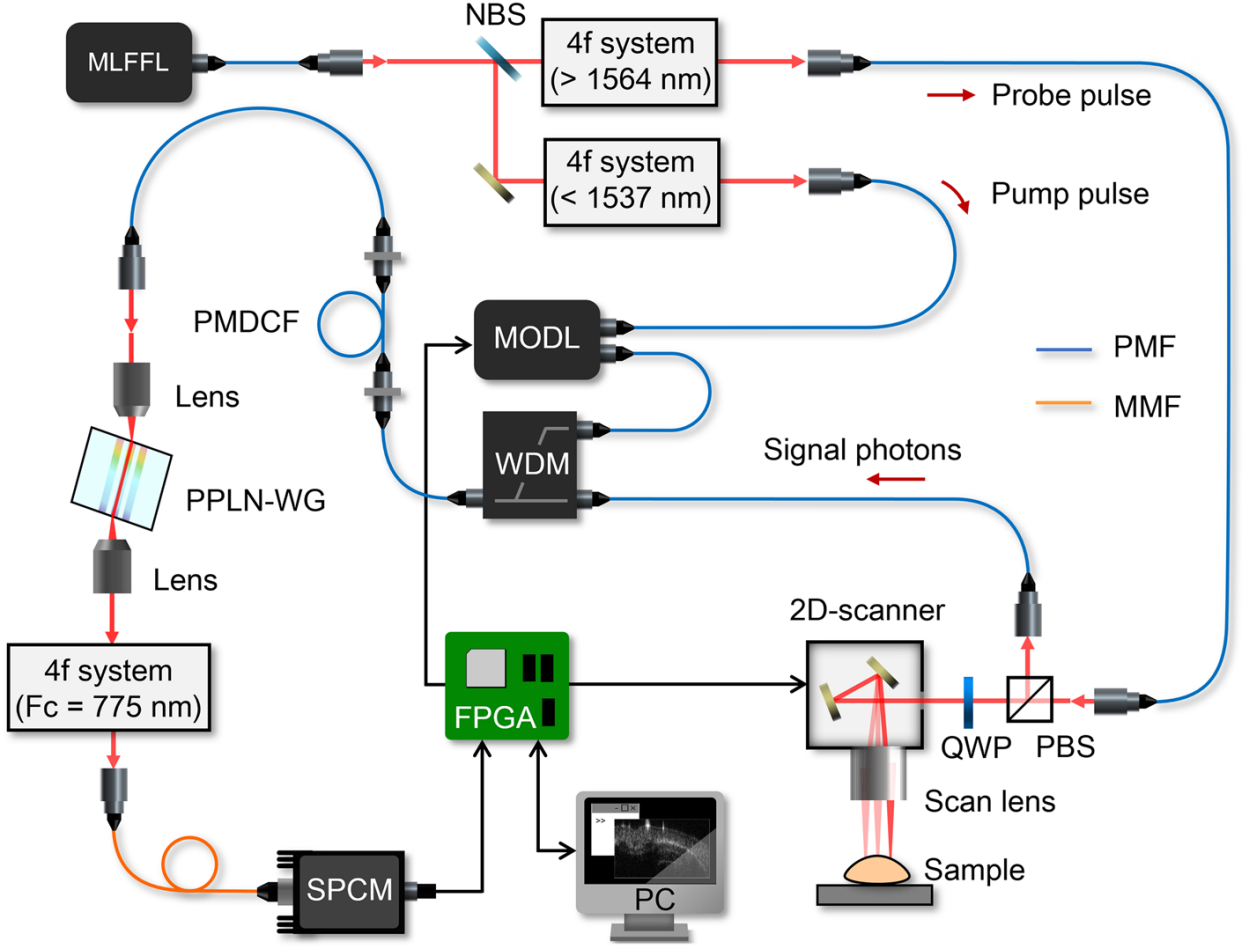
Quantum optical tomography system using a femtosecond up-conversion single-photon detector. Experimental setup. MLFFL, passively mode-locked femtosecond fiber laser; HWP, half waveplate; NBS, non-polarization beamsplitter; QWP, quarter waveplate; PBS, polarization beamsplitter; MODL, motorized optical delay line; WDM, wavelength-division multiplexer; PMDCF, polarization-maintaining dispersion-compensated fiber; PPLN-WG, periodically poled MgO:LiNbO_3_ waveguide; PMF, polarization-maintaining fiber; MMF, multimode fiber; SPCM, single-photon counting module; FPGA, field programmable gate array; PC, personal computer. The three 4f systems are operated as follows. The first one is the wavelength long-pass filter with a cutoff wavelength of 1564 nm. The second one is the wavelength short-pass filter with a cutoff wavelength of 1537 nm. The third one is the wavelength band-pass filter at 775 nm with a bandwidth of 1.5 nm.

The diagram of the experimental setup for quantum optical tomography is illustrated in Fig. 1. A passively mode-locked fiber femtosecond laser (MLFFL: FFL-FR-HP-100 MHz, PriTel) emits 100-fs optical pulses through a 1-m-long polarization-maintaining fiber (PMF) at a center wavelength of 1556 nm with a repetition frequency of 100 MHz. The pulsed laser beam is split into two beams by a non-polarizing beamsplitter (NBS). The transmitted and reflected beams pass through each wavelength filter based on 4f system (detail is described in Methods), and used as probe and pump pulses, respectively. The probe pulses are guided through a PMF to a two-dimensional (2D) scanning system composed of two galvanometer mirrors (GVS001, Thorlabs) and a scan lens (SL50-3P, Thorlabs). They are focused on a measurement sample with a beam waist of ~ 35 μm. To extract the back-scattered signal photons form the sample, an optical circulator composed of a quarter waveplate (QWP) and a polarization beamsplitter (PBS) is used. The signal photons are coupled to a PMF and led to a wavelength-division multiplexer (WDM), where they are combined with the pump pulses. The relative time delay between them is controlled by a motorized optical delay line (MODL: MDL-002, ASM Technology) inserted between the PMFs that lead the pump pulses to the WDM. The total length of PMFs that the probe and the pump pulses respectively pass through is 5 m. The pulse broadening due to the dispersion of PMFs are compensated by a single polarization-maintaining dispersion-compensated fiber (PMDCF). After the compensation, the pulse width of the probe and pump pulses is 380 fs (see Figure 3). Then the signal photons combined with the pump pulse are coupled to a 10-mm-long periodically poled MgO:LiNbO_3_ waveguide (PPLN-WG: AdvR). When the signal photons and the pump pulse satisfy the type-0 phase-matching condition, the frequency of the signal photons ${\nu }_{s}$ is up-converted to ${\nu }_{c}$ (SFG process) according to the energy conservation law: ${\nu }_{c}={\nu }_{s}+{\nu }_{p}$, where ${\nu }_{p}$ is the frequency of the pump pulse. To reject noise photons due to some other nonlinear processes, i.e. second harmonic generation (SHG), difference frequency generation (DFG) and Raman scattering, the up-converted photons are passed through the 4f system that operates as a wavelength band-pass filter at 775.2 nm with a bandwidth of 1.5 nm. Finally, they are coupled to a multimode fiber (MMF) connected to a single-photon counting module (SPCM: SPCM-AQRH-14, Excelitas Technology).

In our tomography system, the 2D horizontal beam scan, the relative time delay, and the photon counting data acquisition are fully controlled using a commercial field programmable gate array (FPGA) board (Zynq UltraScale + MPSoC ZCU104 Evaluation Kit, Xilinx).

### Characteristics of the up-conversion single-photon detector

**Figure 2:**
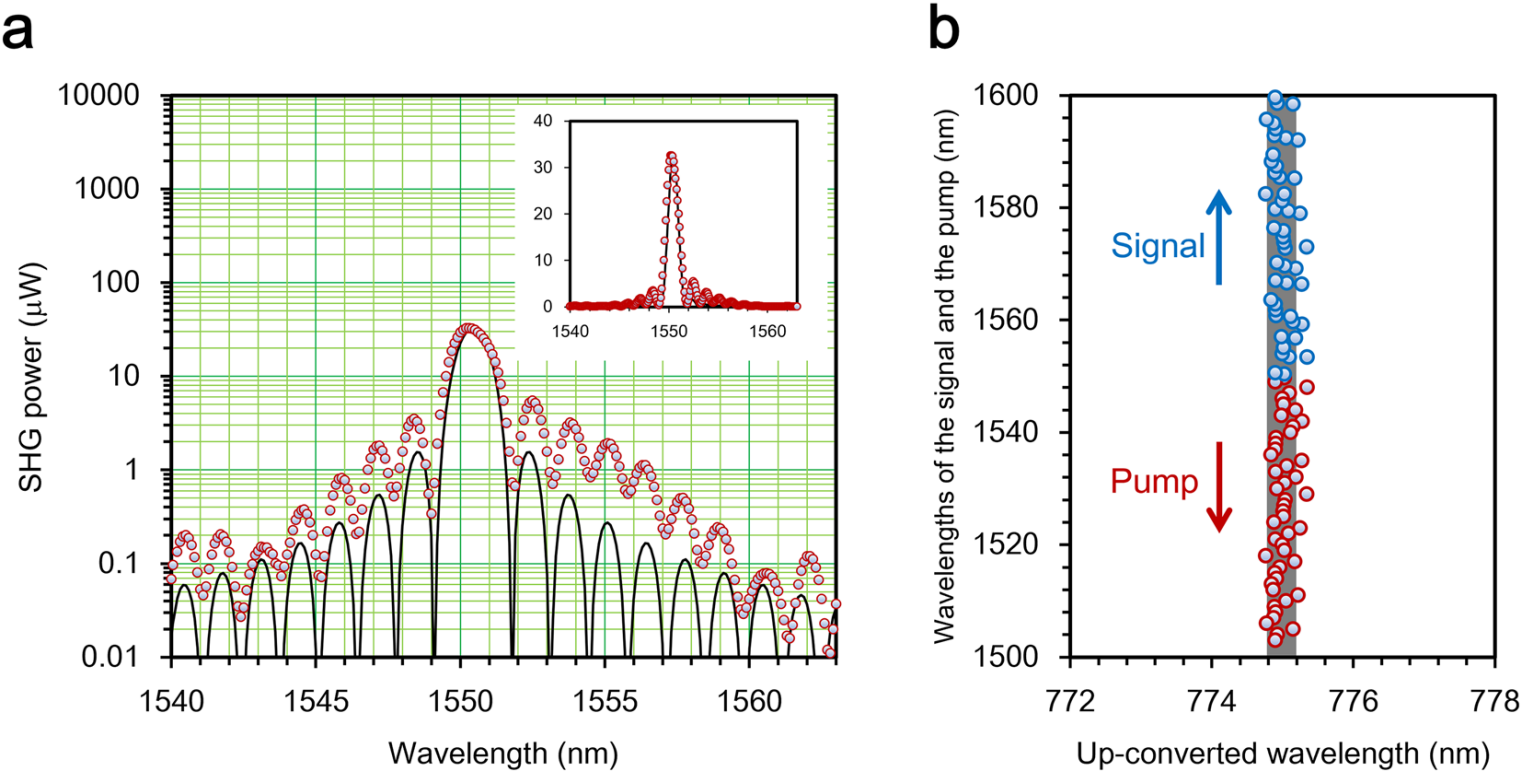
Phase matching characteristics of the 10-mm-long PPLN-WG. (**a**) The second harmonic generation power as a function of the excitation wavelength. The red circles denote measured values and are fitted by an ideal phase matching profile (a black solid curve). The inset shows the same data on a linear scale. (**b**) Measured tuning curve of the 10-mm-long PPLN-WG. The wavelength sets of ${\lambda }_{s}$ and ${\lambda }_{p}$ that satisfy the phase matching condition are plotted as a function of ${\lambda }_{c}$. The set of ${\lambda }_{s}$ and ${\lambda }_{p}$ is found by scanning the pump wavelength for a fixed signal wavelength to maximize the SFG power. Then ${\lambda }_{c}$ was calculated from the energy conservation law ${\lambda }_{c}={\lambda }_{s}{\lambda }_{p}/\left({\lambda }_{s}+{\lambda }_{p}\right)$. The wavelength band within 775.2 $\pm$ 1 nm is highlighted in gray area.

Basic characteristics of the PPLN-WG were carefully evaluated using a wavelength tunable continuous-wave (CW) laser. Figure 2a shows the measured SHG power as a function of the wavelength of the CW laser output coupled to the PPLN-WG at 298 K. The degeneracy wavelength was found to be 1550.4 nm. The wavelength bandwidth of the SHG was evaluated to be 1.30 nm by fitting the experimental data with an ideal phase matching profile $\propto {\left(L/\lambda \right)}^{2}{\mathrm{sinc}}^{2}(\Delta k(\lambda )L/2) \sim {\mathrm{sinc}}^{2} (\mathrm{\alpha \lambda })$, where $\Delta k(\lambda )$ is the phase mismatch between the excitation at $\lambda$ and the SHG at $\lambda /2$, $L$ is the length of the PPLN-WG, and $\mathrm{\alpha }$ is a constant. There is a discrepancy between experimental data and the ideal phase matching profile. It might be caused by a non-uniformity of the periodical poling. The experimental data was fitted by ${\mathrm{sinc}}^{2} (\mathrm{\alpha \lambda })$ so that its period coincides with that of the experimental data to determine the SHG bandwidth. From the SHG power dependence on the coupled laser power (not shown), the normalized conversion efficiency was estimated to be 92%W^−1^ cm^−2^. Here, the Fresnel reflection from input and output facets of the PPLN-WG was taken into account.

To attain the high mode-selectivity as well as the high temporal resolution, the inverse group velocity of the signal photons ${\beta }_{s}$ must be matched to that of the pump pulses ${\beta }_{p}$, which can be investigated via the tuning curve. Figure 2b shows the experimental tuning curve of the 10-mm-long PPLN-WG measured by setting the signal and pump wavelengths using two independent wavelength tunable lasers. It displays wavelength sets $\left\{{\lambda }_{s}, {\lambda }_{p}, {\lambda }_{c}\right\}$ that satisfy the phase matching condition and indicates that the wavelength ${\lambda }_{c}$ has small variance. Here, ${\lambda }_{s}$, ${\lambda }_{p}$, and ${\lambda }_{c}$ are wavelengths of a signal photon, a pump pulse, and a converted photon, respectively. This implies that the inverse group-velocity mismatch (GVM) between the signal and pump pulses negligibly small (theoretical detail is described in Methods), and the “near-zero” GVM was ensured over more than 100 nm. According to the theoretical calculation using the refractive index of a 5 mol.%-MgO-doped lithium niobate bulk crystal, the GVM between the signal photons and the pump pulse ${\beta }_{s}-{\beta }_{p}$ is as small as 2.5 fs $\cdot$ mm^−1^. Although the waveguide dispersion may change the GVM slightly, it would be much shorter than the temporal width of the pump pulse (380 fs) used in our experiments.

**Figure 3:**
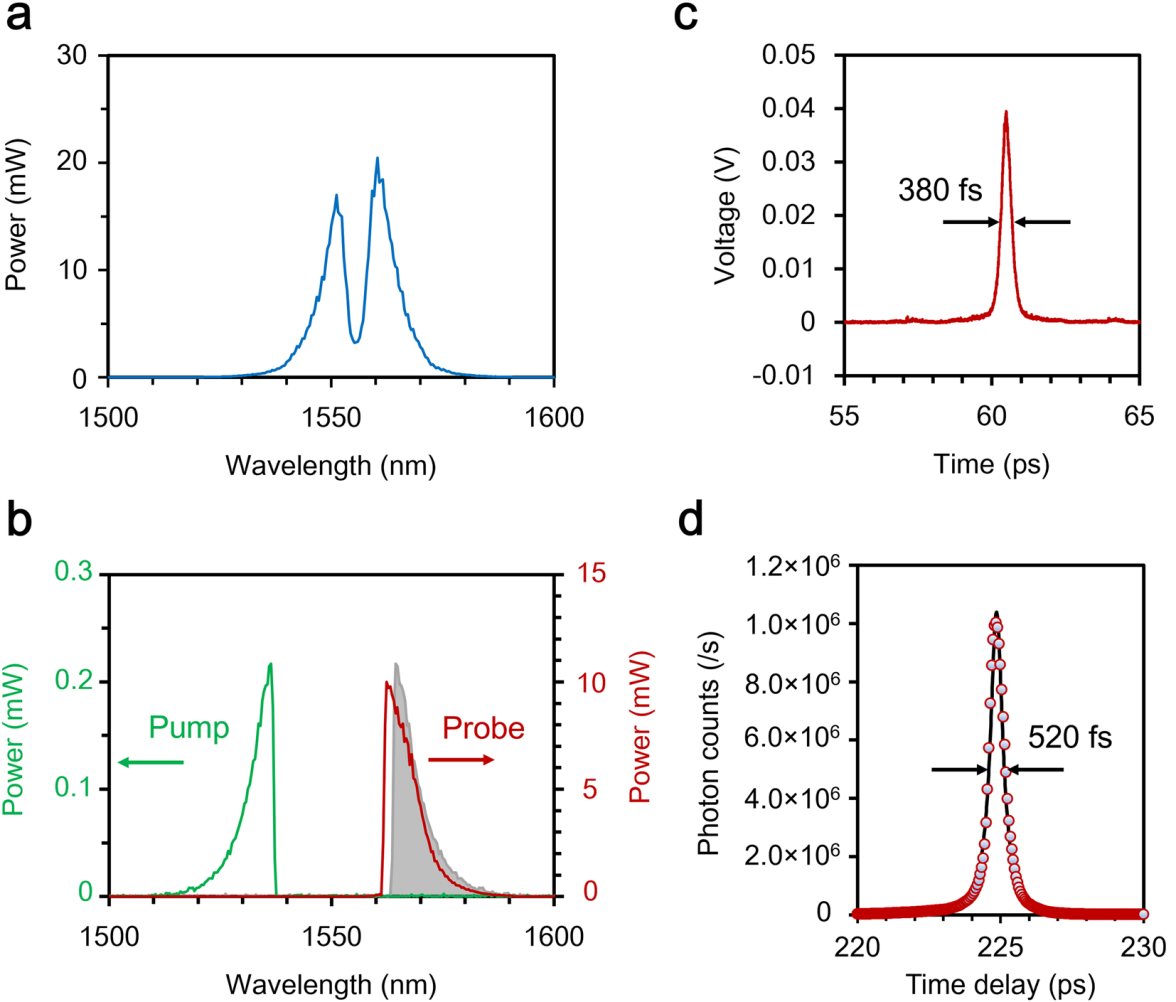
Characteristics of the probe and pump pulses. (**a**) The spectrum of the MLFFL output through a 1-m-long PMF. (**b**) The spectra of the pump and probe pulses. The gray shadow denotes the conversion spectrum which is calculated by the spectrum of the pump pulse and the phase matching relation. Photons spectrally overlapped with it is efficiently up-converted. (**c**) The autocorrelation trace of the probe pulse dispersion-compensated by the PMDCF. (**d**) A temporal profile of the probe pulse reflected by a gold mirror which was replaces with the measurement sample.

The Fig. 3a shows the spectrum of the MLFFL through a 1-m-long PMF. To obtain the probe pulse (the pump pulse), we used the 4f system that operates as the long-wavelength-pass (short-wavelength-pass) filter with a cutoff wavelength of 1564 nm (1537 nm). The spectra of the probe and pump pulses are shown in Fig. 3b. They have almost the same spectral bandwidth of 740 GHz (the full width at half maximum, FWHM) or 6 nm in wavelength. They look like a one-sided exponentially decaying function. Therefore their temporal shapes would be approximated by Lorentzian rather than Gaussian. We assume Lorentzian pulses for the pump and probe to numerically calculate the mode separability and selectivity under our experimental conditions. Although the optical power of the pump pulse (< 0.1 mW) is too low to measure its temporal shape by an autocorrelator, the probe pulse has enough power (> 1 mW) to measure it. According to the autocorrelation trace shown in Fig. 3c, the temporal width of the probe pulse is 380 fs. The temporal width $\Delta {T}_{p}$ of the pump pulse is assumed to be the same as that of the probe pulse.

The temporal resolution of the time-of-fright measurement system shown in Fig. 1 can be evaluated replacing the measurement sample with a gold mirror. The maximal SFG power 1.14 μW (after the MMF coupling) was obtained when the relative time delay between the pump and the signal pulses was set to zero corresponding to 224.86 ps in Fig. 3d. The SFG power easily makes the SPCM blind. Therefore, the signal pulses were attenuated by 70 dB in order to evaluate the temporal resolution. Figure 3d shows photon count rates as a function of the relative time delay given by the MODL. The temporal width was 520 fs which correspond to the axial resolution of 78 μm in air. Assuming the refractive index of a mouse brain to be 1.37^29,30^, the axial resolution would be 57 μm.

As mentioned above, the SFG peak power is 1.14 μW. On the other hand, the noise equivalent power (NEP) given by $h\nu {\eta }^{-1}\sqrt{2{R}_{N}}$ is $1.0 \times 10^{ - 17}$ W/Hz^1/2^ assuming that the up-converted photons are detected by the SPCM, where $h$ is the Planck constant, $\eta$ is a single-photon detection efficiency (0.65 at 775.2 nm), and ${R}_{N}$ is a noise count rate (1500 counts/s: dark counts and detected noise photons) of the SPCM. When the integration time is one second, the sensitivity of our quantum tomography system is as high as 111 dB that exceeds the typical sensitivity of OCT by more than 10 dB. Here, the sensitivity is defined as the maximum allowable optical losses imposed on the probe pulse by a specimen. It is considered equivalent to the signal-to-noise ratio (SNR) without the losses. We note that the sensitivity was achieved with the average power of 30 μW for the pump (coupled to the PPLN-WG) and 1.5 mW for the probe (before the measurement sample).

### Analysis of the mode separability and selectivity

To assess the performance of our mode-selective up-conversion single-photon detector, we investigated how effectively the signal component carrying target information can be extracted from the total radiation field contaminated by multiply scattered photons. To this end, we performed numerical simulations of the three-wave mixing frequency up-conversion process and calculated the two-time Green function, or more precisely, the signal-idler transfer function ${G}_{rs}\left(t, {t}^{\prime}\right)$^21^. (We note that the “idler” should be read as the “converted photons” in this study, but we preserve this term in this section for consistency with previous theoretical studies.)

**Figure 4:**
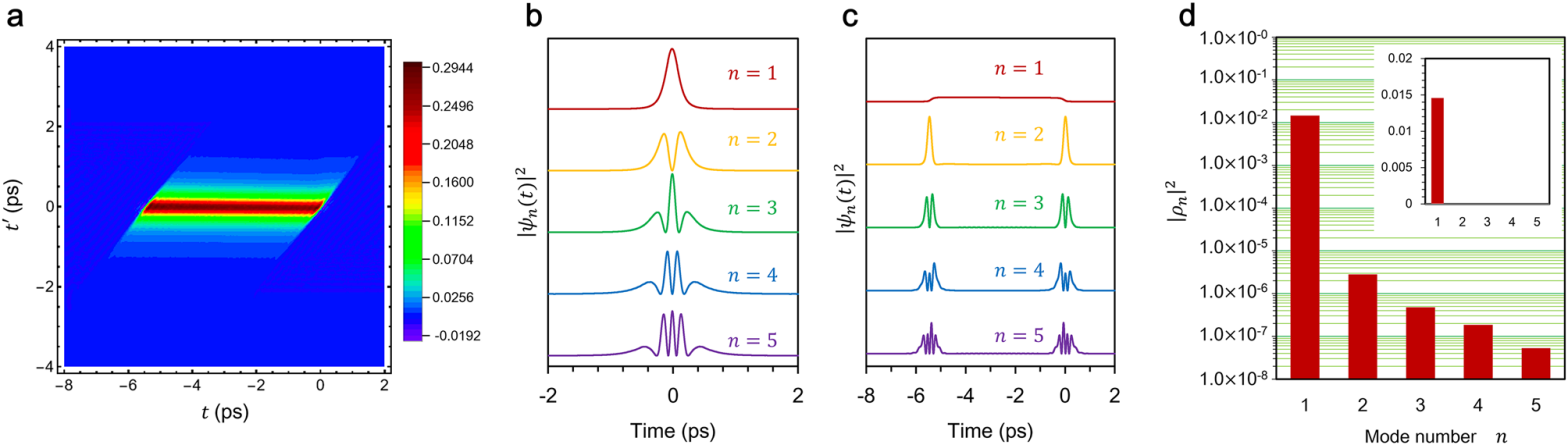
Two-time transfer function, numerical Schmidt mode profiles and conversion efficiencies. (**a**) The contour plot of the two-time transfer function ${G}_{rs}\left(t, {t}^{\prime}\right)$ under the condition that ${\beta }_{s}-{\beta }_{p}=2.5$ fs/mm, ${\beta }_{c}-{\beta }_{p}=-0.55$ ps/mm, and $\gamma =0.0825$ mm^-1^. (b) Temporal profiles of five leading Schmidt modes of the signal. (**c**) Temporal profiles of five leading Schmidt modes of the idler. (**d**) Conversion efficiencies ${\left|{\rho }_{n}\right|}^{2}$ of five leading Schmidt modes.

The Fig. 4 shows ${G}_{rs}\left(t, {t}^{\prime}\right)$ calculated using the parameter values estimated from experimental results, as well as the five leading Schmidt modes and conversion efficiencies obtained by decomposing the function. Since the pump and probe pulses in our experiment are spectrally carved from the mode-locked fiber laser using the 4f system as shown in Fig. 3b, their temporal waveforms cannot be Gaussian but rather Lorentzian. Therefore, we assumed a Lorentzian waveform for the pump and probe pulses in our simulations. The temporal waveform of the Schmidt mode is strongly influenced by that of the pump pulse, because the conversion occurs only when the signal pulse overlaps temporally with the pump pulse. In our experiment, the coupling strength ($\gamma =$ 0.0825 mm^−1^) is small, and the GVM between the pump and the idler pulses (${\beta }_{c}-{\beta }_{p}=-0.55$ ps $\cdot$ mm^−1^) is relatively large (details are described in ‘Methods’ section). This latter condition ensures that the idler pulse, once created, departs from the pump pulse promptly without further interaction. Consequently, these conditions allow the principal Schmidt mode to be a “replica” of the pump pulse with a Lorentzian waveform, which guarantees that the temporal mode of the signal pulse favorably matches the principal Schmidt mode as displayed in Fig. 4b.

The temporal width (FWHM) of the pump pulse $\Delta {T}_{p}$ is a crucial yet easily adjustable parameter. For our experiment, we chose $\Delta {T}_{p}$ to be equal to that of the signal pulse $\Delta {T}_{s}$ = 380 fs. As mentioned earlier, the principal Schmidt mode closely resembles the pump pulse shape under the current experimental conditions. Thus, if the signal pulse waveform ${A}_{s}(t)$ is expanded using the Schmidt modes $\left\{{\psi }_{n}\right\}, \left(n=1, 2, \dots \right)$ as ${A}_{s}\left(t\right)={\sum }_{n}{c}_{n}{\psi }_{n}(t)$, the expansion is dominated by the leading term (the principal Schmidt mode) with only a small contribution from the other terms, as long as $\Delta {T}_{p}\cong \Delta {T}_{s}$. If this condition is not satisfied, then higher Schmidt modes could significantly contribute to the signal pulse and reduce the selectivity of the frequency conversion. However, as shown in Fig. 4d, the Schmidt coefficients for higher modes are negligibly small compared to that of the principal Schmidt mode, indicating that the transfer function is highly separable^31^. This suggests that the choice of the pump pulse width is quite robust in our experimental setup, as the converted photons are primarily generated from the principal Schmidt mode even when the signal pulse involves other modes.

**Figure 5:**
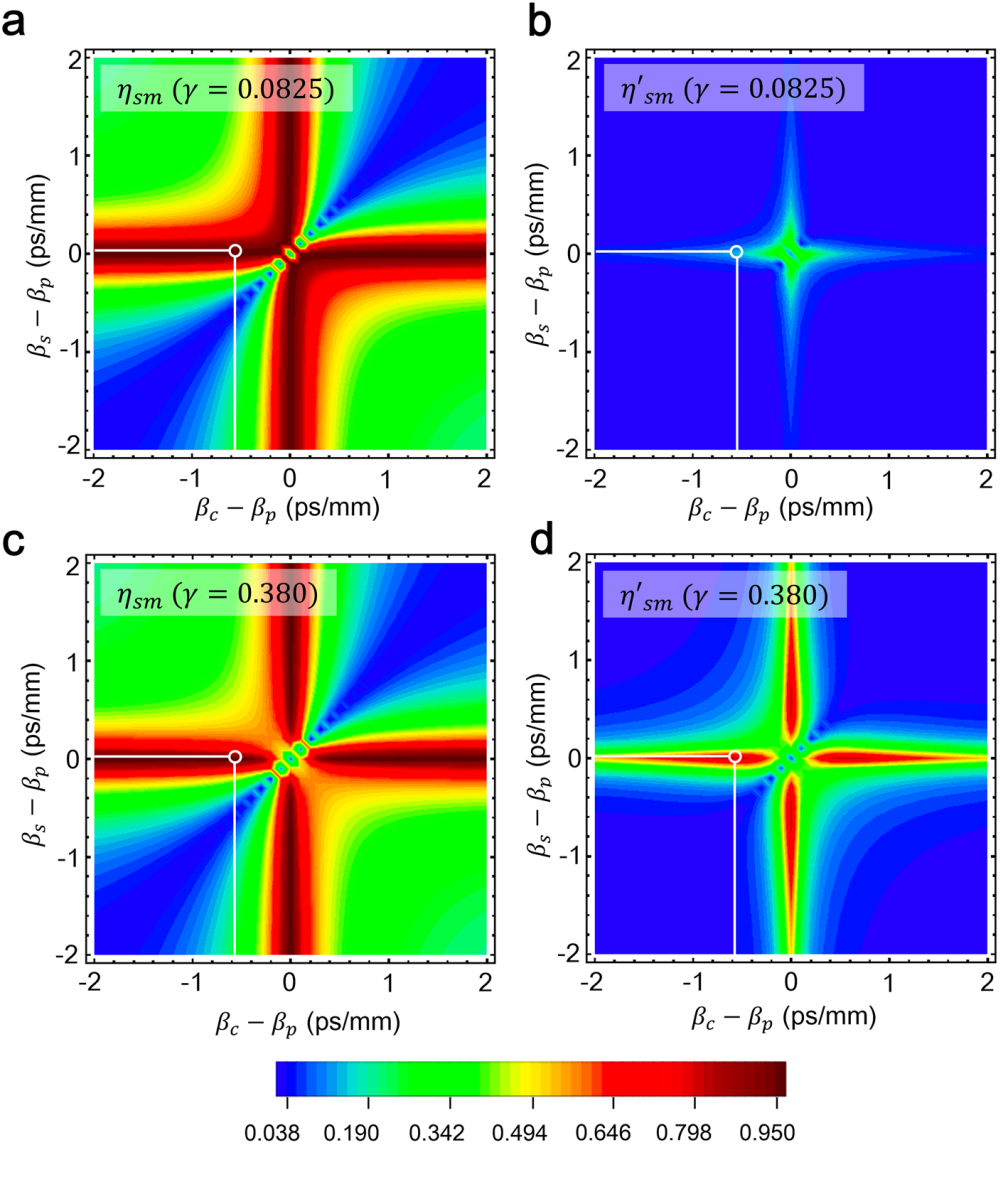
Numerical mode separabilities and selectivities for $\gamma =0.0825$ mm^−1^ and $\gamma =0.38$ mm^−1^.

The product of the temporal width of the pump pulse and the phase matching bandwidth $\Delta {T}_{p}\Delta B$ is calculated to be 6.1$\times$10^−2^ that is much smaller than unity, and the GVM between the pump and signal pulses is negligibly small. This indicates that only the singly reflected signal photons will be efficiently up-converted if their temporal modes are matched to the principal Schmidt mode shown in Fig. 4b. According to our numerical results in the low conversion regime, the principal Schmidt mode of the signal pulse is a Lorentzian shape. Therefore, the Lorentzian pump pulse used in our experiment would effectively up-convert the signal photons. Even if their pulse broadening due to the dispersion of the mouse brain is not negligibly small, other modes will not be converted as shown in Fig. 4d. The mode separability ${\eta }_{sm}$ is defined as

1 $${\eta}_{sm} { = }\frac{{\left| {{\rho}_{n} } \right|^{2} }}{{\sum\nolimits_{n} {\left| {{\rho}_{n} } \right|^{2} } }}{,}$$

where, ${\rho }_{n}$ is the coefficient of the Schmidt mode ${\psi }_{n}$ of the signal photons. The numerically calculated ${\eta }_{sm}$ with $\gamma$ = 0.0825 as a function of ${\beta }_{c}-{\beta }_{p}$ and ${\beta }_{s}-{\beta }_{p}$ is shown in Fig. 5a. The principal Schmidt mode ${\psi }_{1}$ is selectively up-converted and the other modes ${\psi }_{n\ne 1}$ is barely converted. As a result, the mode separability as high as 0.99 can be achieved. Since the up-conversion efficiency is only 11%, the mode selectivity defined as

2 $${\eta}_{sm}^{\prime} = \frac{{\left| {{\rho}_{n} } \right|^{4} }}{{\mathop {\sum }\nolimits_{n}^{{}} \left| {{\rho}_{n} } \right|^{2} }}{,}$$

is 0.11 as shown in Fig. 5b. By increasing the pump power, the up-conversion efficiency can be enhanced, which results in the larger selectivity. The mode selectivity was numerically calculated with various pump power. Figure 5c,d shows numerical results of the separability and selectivity when $\gamma =$ 0.38. Thanks to the very small GVM between the signal photons and the pump pulse (2.5 fs/mm), the mode selectivity of 0.79 will be achieved while keeping the mode separability of 0.98 as shown in Fig. 5c. We note that only the coupled average pump power of 600 μW (a temporal width of 380 fs and a repetition frequency of 100 MHz) will be required to achieve the highest mode selectivity.

### Mouse brain imaging

**Figure 6:**
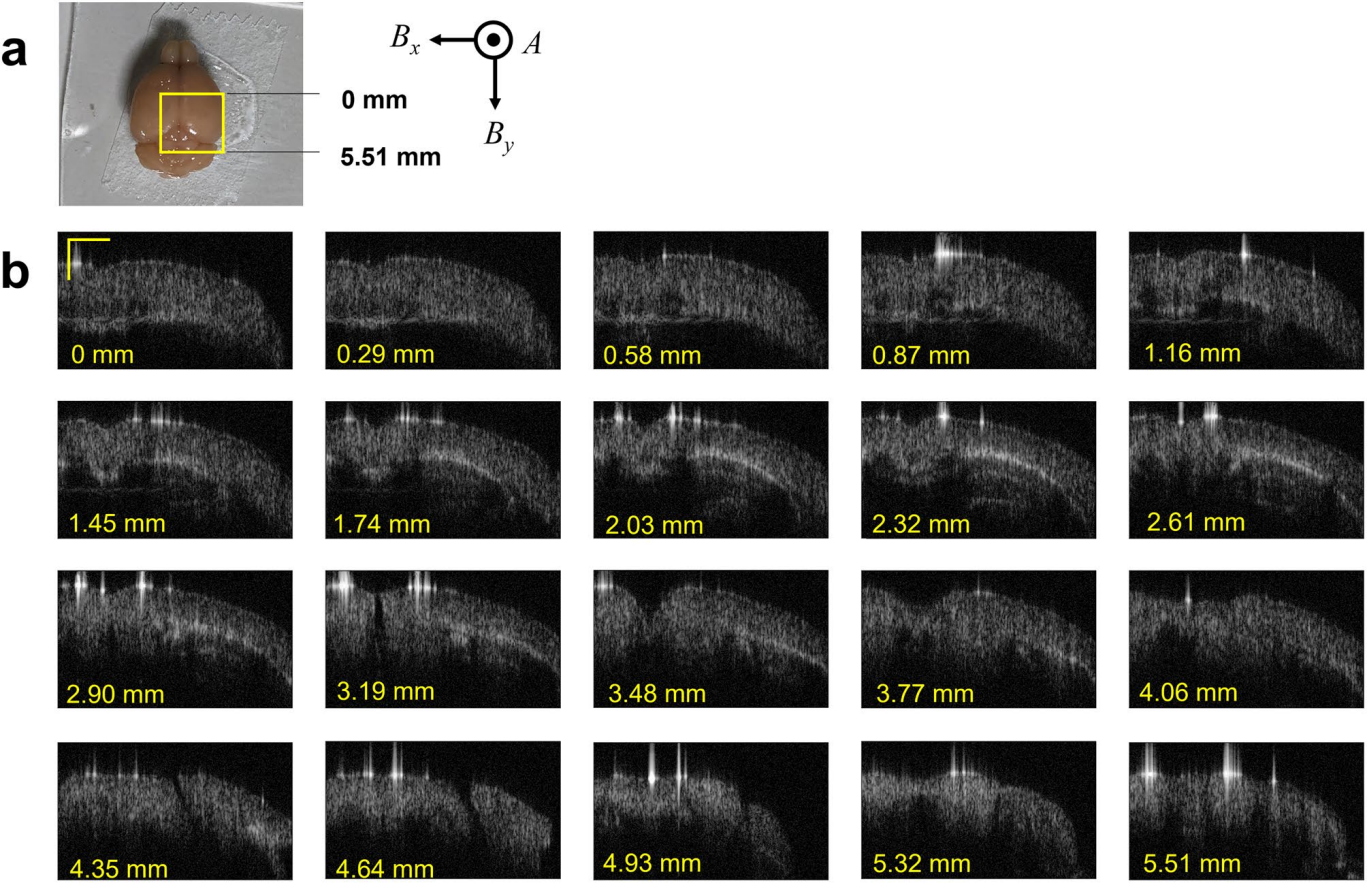
Perfusion-fixed mouse brain and the tomographic images. (**a**) The 5.51 $\times$ 5.51-mm spatial area of a perfusion fixed mouse brain shown by a yellow square was scanned in the lateral direction. The line scans in the *x* and *y* directions are defined as *B*_*x*_ scan and *B*_*y*_ scan, respectively. The line scan in the depth direction is defined as *A* scan. (**b**) *A-B*_*x*_ scan coronal images at various depths from the rostral end of the imaging field. The bright (white) layer extending laterally in the brain is inferred to be the lipid-rich corpus callosum. Scale bar: 1.0 mm.

The Fig. 6 shows the examples of 2D-images of a perfusion-fixed mouse brain (See Methods). The scanning ranges in the lateral *x* and *y* directions (*B*_*x*_ and *B*_*y*_ scan) are 5.51 mm, and the scanning area (*B*_*x*_ − *B*_*y*_) is shown in Fig. 6a. The scan in the axial or depth direction (*A* scan) was performed with a step of 100 fs (10.6 μm in depth). The imaging format is 256 $\times$ 512 (*A* scan $\times$ *B*_*x*_ scan) and the scan rate is set to be 3 Hz for the *B*_*x*_ scan. Therefore, the NEP of the SPCM was degraded by the square root of (3 $\times$ 512) and consequently the sensitivity was reduced to 95 dB. The sensitivity is still competing with that of the current OCT. The coronal images at *B*_*y*_ = 1.74 to 3.19 mm in Fig. 6b demonstrate the bright white layer extending laterally. It corresponds to the myelin lipid-rich corpus callosum and the alveus of hippocampus. Beneath the corpus callosum, faint but clear white lines are confirmed at *B*_*y*_ = 2.03 and 2.32 mm. They might be the upper layer of the hippocampal dentate gyrus and the brachium of the superior colliculus, respectively. In the cortical layers, relatively uniform signals are detected, which implies that they have similar scattering properties despite their cytoarchitectural differentiation. Signal intensities from these brain structures seemed to reflect the amount of myelin.

**Figure 7:**
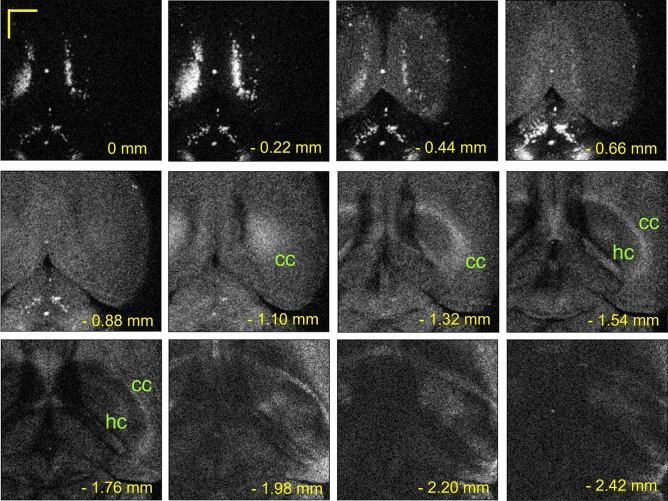
*B*_*x*_ − *B*_*y*_ scan images in various depths from the brain cortical surface. At the depth of 0 ~ 1.98 mm (2.20 mm and 2.42 mm), images were acquired with a *B*_*x*_ scan rate of 3 Hz (1 Hz). cc: corpus callosum, hc: hippocampus. Scale bar: 1.0 mm.

Next, fixing the relative time delay given by the MODL, the lateral-2D imaging was carried out. Figure 7 shows the *B*_*x*_ − *B*_*y*_ scan images at various depths below the brain cortical surface with 512 $\times$ 512 format. The scan area is the same as that shown in Fig. 6a. The bilateral bright area in the cortex at the depth of 0 to 2.42 mm in Fig. 7 shows the retrosplenial cortex that received highly myelinated axonal fibers. The bright elliptical line shown at the depth of 1.32 to 2.20 mm in Fig. 7 is inferred to be the corpus callosum (cc) and the alveus of the hippocampus (hc). The brain structures are imaged in detail with the much better contrast comparing to the *A*-*B*_*x*_ scan images. This might be ascribed to the accuracy and the repeatability of the MODL used in our experiments.

## Discussion

In the OCT system, weak signals are amplified by the interference with the relatively strong reference light wave. This is the reason why the sensitivity of OCT is in principle governed by irreducible quantum field (vacuum) fluctuations amplified by the reference light wave (so-called shot noise)^33^. The shot-noise power is proportional to the reference light power ${P}_{r}$. On the other hand, the OCT output power is proportional to ${P}_{p}{P}_{r}$, where ${P}_{p}$ is the probe light power. Interestingly, the shot-noise-limited sensitivity defined as the ratio of the OCT output power without a specimen to the shot-noise power does not depend on ${P}_{r}$. The photodetector noise and the relative intensity noise (RIN) also degrade the sensitivity of OCT. Recently, the shot-noise limited OCT has been demonstrated and its sensitivity was ~ 105 dB because the probe light power is restricted to several milliwatts^34,35^, which indicates that the maximal penetration depth of OCT cannot be improved without increasing the probe light power. Even if the single-photon detector (or single-photon camera) is employed to efficiently detect the OCT signal with substantially low noise counts, the sensitivity is still limited by the shot noise^36,37^. However, the use of the single-photon detector allows us to strongly attenuate the reference light wave, which results in the suppression of its excess intensity noise that inhibits the shot-noise-limited detection of the OCT signal.

On the other hand, the sensitivity of our quantum optical tomography system is simply determined by the SNR of the up-conversion single-photon detection. By improving the efficiency of the entire system, the sensitivity will be enhanced. The optical loss of the current system can be reduced by more than 10 dB improving the coupling efficiency from free-space to PPLN-WG with antireflection-coatings and replacing the gratings in 4f systems with more efficient ones. The noise count rate of the SPCM ${R}_{N}$ includes the detected noise photons mainly due to Raman scattering induced by the pump pulse in PMFs. They must have been removed by considerable amount via the mode-selective up-conversion, otherwise the pump pulse whose wavelength was only 30 nm away from the signal pulse wavelength would drastically increase the noise counts^26^. The noise photons were only one order of magnitude higher than the dark counts of the SPCM (100 counts per second). They can be eliminated if a free-space type of a WDM coupling system with an edge pass filter is employed. Therefore, the ${R}_{N}$ would be reduced by more than 10 dB (> threefold SNR enhancement). The time gating is also beneficial to the reduction of dark counts. For example, 1-ns gate for the single-photon detection (100 MHz repetition) can easily reduce the dark counts (except for noise photons) by 10 dB. Furthermore, increasing the pump power up to 600 μW, the up-conversion efficiency can be enhanced by 8 dB (See Fig. 5d). Thus, a sensitivity of more than 140 dB would be achievable using probe pulses with an average power of 5 mW which is below the ANST standard for the maximally allowed laser power^38^.

The OCT would be difficult to achieve a high sensitivity with the restricted probe power. In the shot-noise limited OCT, the minimum detectable photon flux ${\Phi }_{m}^{OCT}$ (photons per second) is given by $2B/\eta$, where $\eta$ is the detector’s quantum efficiency and $B$ is the Nyquist bandwidth corresponding to the measurement time ${\tau }_{m}$^33,37^. If we assume that $\eta =1$ (ideal case), ${\Phi }_{m}^{OCT}=2B=1/{\tau }_{m}$. It means that we have to detect one photon during ${\tau }_{m}$ to achieve SNR = 1. On the other hand, in our photon counting case, the average noise counts during the measurement time are ${R}_{N}{\tau }_{m}$. They must be one to achieve SNR = 1 with the same photon flux (analyzed from the NEP of the photon detection). If ${\tau }_{m}$ is assumed to be 1 ms, the corresponding ${R}_{N}$ is 10^3^. ${R}_{N}$ in the present experiment is already comparable to this value, and it can be suppressed in the manner described above. Therefore, our tomography system can achieve much higher sensitivity than the shot-noise limited OCT system.

The improvement of the efficiency described above also allows us to boost the scanning rate, because the flux of up-converted photons is drastically increased. The 30 dB improvement of the sensitivity corresponds to three orders of magnitude enhancement of the photon flux. Therefore, the scan rate can be increased up to several kilohertz, corresponding to 10 frame per second for 2D imaging. However, we note that the sensitivity will be degraded because the noise power depends on the bandwidth. Therefore, the NEP of a single-photon detector is proportional to a square root of the bandwidth. Then the sensitivity will be degraded by 15 dB, but at least 125 dB sensitivity is still available.

Currently the axial resolution of our system is limited by the relatively wide temporal widths (380 fs) of the pump and probe pulses. Utilizing the-state-of-the-art mode-locked femtosecond fiber laser and the supercontinuum generation technology, the pump and probe pulses with a temporal width of several tens of femtoseconds (or a spectrum broader than 100 nm) can be generated. Therefore, the axial resolution less than 10 μm would be achieved. Here, the axial-resolution inherent in the up-converter must be taken into account. The GVM between the signal and pump pulses in the PPLN-WG is negligibly small but not zero. The pump pulse is delayed by 25 fs relative to the signal pulse over the 10-mm-long PPLN waveguide, which affects the temporal resolution when the temporal width of the signal and the pump is set to less than 67 fs corresponding to axial resolution of 10 μm in air.

In the present experiment, we demonstrated tomographic imaging based on the fundamental-mode-selective and time-resolved photon detection. Tailoring the temporal mode of the pump pulse, the arbitrary-mode-selective photon detection can be achieved, which is beneficial to the further suppression of detecting noise photons. This is the important step towards the high-contrast optical imaging of deeper complex structures in biological samples.

Finally, we mention about the system cost. Our optical setup is similar to that of the OCT system except for the photon detection setup. The system-cost increase that comes mainly from the PPLN-WG and the single-photon counting module is not so high. Images of our system are simply obtained from the photon counts without complex reconstruction processes, which indicates that the computational cost is very low compared with the OCT systems.

## Conclusion

We have developed the quantum optical tomography system based on the time-resolved and mode-selective single-photon detection and demonstrated 111 dB sensitivity with a probe pulse average power of 1.5 mW. The sensitivity is comparable to that of the shot-noise-limited OCT system. Although the axial resolution in our current system is 57 μm, less than 10 μm resolution is feasible by employing probe and pump pulses whose pulse widths are several tens of femtoseconds. In addition, more than 140 dB sensitivity would be achievable by increasing pump powers as well as reducing the optical losses and noise photons in the current system. We applied our tomography system to acquire structural information of a mouse brain and demonstrated high contrast 2D tomography up to ~ 2 mm in depth. The cerebral cortex gray matter exhibited uniform scattering characteristics. Therefore, the tomography system might be suitable for early-stage detection of brain diseases characterized by the aggregation of highly scattering substances in the cortex in vivo through longitudinal assessments^39^. Our quantum optical tomography system will open a new way of non-contact and non-invasive three-dimensional structural imaging of not only biological specimens but also strongly light-scattering materials.

## Methods

### Animals

All procedures and experiments were performed in accordance with ARRIVE guidelines. All animal experiments were conducted in accordance with the National Institutes of Health Guide for Care and Use of Laboratory Animals (NIH Publications No. 8023) and approved by the Animal Ethics Committee of Keio University (approval number: A2022-299). All methods were carried out in accordance with relevant guidelines and regulations. Five adult male C57BL/6 J mice purchased from SLC (Shizuoka, Japan) were deeply anesthetized and transcardially perfused with phosphate buffered saline (PB) containing 4% paraformaldehyde (PFA). The brains were removed and postfixed in 4% PFA at room temperature overnight. PB was used during imaging.

### Wavelength filter based on 4f system

**Figure 8:**
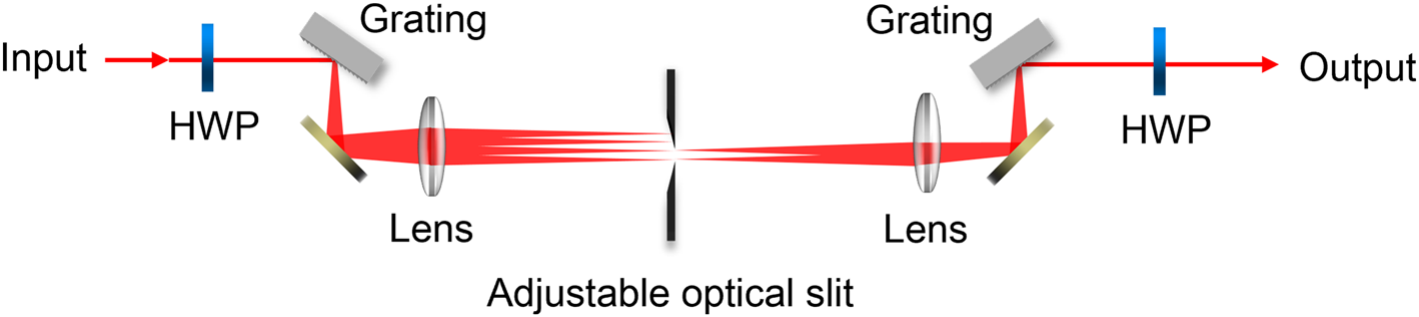
The schematic diagram of the 4f system. HWP, half waveplate.

The 4f system consists of a pair of gratings, a pair of lenses with a focal length $f=$ 200 mm, and an adjustable optical slit as shown in Fig. 8. The lenses are separated by $2f$ and the gratings are placed on the (back) focal planes of each lens. By placing the adjustable optical slit on the Fourier plane, the system operates as a tunable wavelength filter which adjusts both a center wavelength and a bandwidth. The 4f system has two specific features: the very sharp roll-off (cut-off) characteristics and the very high stability of spatial-mode profile over the system. The former is essential for reducing unnecessary spectral components that may introduce noise photons, and the latter is important for efficient coupling from free-space to a PMF. The pulses filtered by the short-pass (long-pass) 4f system are used as the pump (probe). To optimize the efficiency (transmittivity) of the 4f systems, the polarization of the pulsed laser beam is adjusted to be horizontal in advance using a half-wave plate (HWP).

### Group-velocity matching

The tuning curve of the periodically poled second-order nonlinear crystal is given by the energy and the momentum conservation laws:

3 $$\frac{1}{{{\lambda}_{c} }} = \frac{1}{{{\lambda}_{s} }}+\frac{1}{{{\lambda}_{p} }},$$

4 $$\frac{{n\left( {{\lambda}_{c} } \right)}}{{{\lambda}_{c} }}=\frac{{n\left( {{\lambda}_{s} } \right)}}{{{\lambda}_{s} }}+\frac{{n\left( {{\lambda}_{p} } \right)}}{{{\lambda}_{p} }}+\frac{1}{{\Lambda}},$$

where ${\lambda }_{s}$, ${\lambda }_{p}$, and ${\lambda }_{c}$ are wavelengths of a signal photon, a pump pulse, and a converted photon, respectively, $n\left(\lambda \right)$ is the refractive index at a wavelength of $\lambda$, and $\Lambda$ is the poling period of the PPLN-WG. If the wavelength of the converted photon ${\lambda }_{c}$ scarcely depends on ${\lambda }_{s}$ or ${\lambda }_{p}$ as shown in Fig. 2b , the following equation holds:

5 $$\frac{d}{{d\lambda_{s} }}\left[ {\frac{{n\left( {{\lambda}_{s} } \right)}}{{{\lambda}_{s} }}+\frac{{n\left( {{\lambda}_{p} } \right)}}{{{\lambda}_{p} }}} \right] = \frac{d}{{d\lambda _{s} }}\left[ {\frac{{n\left( {{\lambda}_{c} } \right)}}{{{\lambda}_{c} }} - \frac{1}{{\Lambda}}} \right] = 0 .$$

Then, the simple calculation shows that the inverse group velocity of a signal photon ${\beta }_{s}$ is matched to that of a pump pulse ${\beta }_{p}$:

6 $$\frac{d}{{d\lambda_{s} }}\left[ {\frac{{n\left( {{\lambda}_{s} } \right)}}{{{\lambda}_{s} }}+\frac{{n\left( {{\lambda}_{p} } \right)}}{{{\lambda}_{p} }}} \right] = {\beta}_{s} - {\beta}_{p} = 0 ,$$

where ${c}_{0}$ is the speed of light in vacuum and ${\beta }_{k}=\left\{-{\lambda }_{k}\left(dn\left({\lambda }_{k}\right)/d{\lambda }_{k}\right)+n\left({\lambda }_{k}\right)\right\}{{c}_{0}}^{-1} \left( k=s,p\right)$.

### Phase matching bandwidth

The phase matching bandwidth $\Delta B$ can be derived from the phase matching profile shown in Fig. 2a. Although the temporal modes of the signal and pump pulses are considered to be Lorentzian, the up-converted pulses are temporally broadened and they have a rectangular shape (see Fig. 4c) because of the temporal walk-off due to the GVM between the pump and up-converted pulses. Assuming the up-converted pulses are Fourie transform limited, their frequency bandwidth (sinc^2^ profile) would be equal to the phase matching bandwidth $\Delta B$. The relationship between the temporal pulse width $\Delta t$ and the frequency bandwidth $\Delta B$ of a rectangular pulse is given by

7 $$\Delta t\Delta B = \Delta t\Delta \lambda \left( {\frac{{dB}}{{d\lambda}}} \right) = {\Delta t\Delta \lambda }\frac{c}{{{\lambda}^{2}}}=0.88 .$$

The temporal width $\Delta t$ is determined by the group delay over the PPLN-WG:

8 $$\Delta t = L\left( {{\beta}_{c} - {\beta}_{0} } \right),$$

where we assume that the difference between group velocities of the signal and pump pulses is negligibly small: ${\beta }_{s} \sim {\beta }_{p}={\beta }_{0}$. Substituting the measured $\Delta \lambda =$ 1.30 nm to Eq. (7), $\Delta B$ and $\Delta t$ are calculated to be 160 GHz and 5.5 ps, respectively. Then the GVM between the pump and up-converted pulses is $-$ 0.55 ps $\cdot$ mm^−1^ using Eq. (8).

### Coupling strength

The ${\gamma}$ parameter characterizes the coupling strength between the signal and pump pulses. Here, ${\gamma}={D}\sqrt{8{\pi}^{2}{{d}_{eff}}^{2}{P}_{p}/\left({n}_{s}{n}_{p}{n}_{c}{c}{\epsilon}_{0}{{\lambda}_{s}}^{2}\right)}$, where ${d}_{eff}$ is an effective nonlinear coefficient, ${P}_{p}$ is an average peak power of the pump pulse coupled to the PPLN-WG, ${\epsilon}_{0}$ is the permittivity of free space, and ${D}$ is a spatial overlapping coefficient^40^. The value of ${\gamma}$ can be estimated to be 0.0825 mm^-1^ from the normalized conversion efficiency 92%W^−1^ cm^−2^ (~ $100\cdot{\gamma}^{2}{{P}_{p}}^{-1}$) and the average peak power of the pump pulse 0.74 W. Assuming ${\gamma}=$ 0.0825 mm^−1^, ${\beta}_{c}-{\beta}_{p}=-0.55$ ps $\cdot$ mm^−1^, ${\beta}_{s}-{\beta}_{p}=$ 2.5 fs $\cdot$ mm^−1^, and the temporal width of the signal and pump pulses is 380 ps, the conversion efficiency is numerically calculated to be 12%. This value is in good agreement with the measured conversion efficiency of 11% for pulses.

### Computational method

Our simulations of various input signal waveforms resulted in the two-time transfer function ${G}_{rs}(t, t^{\prime})$, which converts an arbitrary signal pulse at the input-side edge of the nonlinear crystal into an output idler pulse (up-converted photons) at the opposite end. Applying the Schmidt decomposition to this transfer function yields the Schmidt modes and Schmidt coefficients^32^. The principal Schmidt mode, with the largest Schmidt coefficient, represents the waveform of the signal pulse that can be converted into an idler pulse with the highest selectivity^21^. Thus, when the temporal mode of the signal photons is similar to or approximates this principal Schmidt mode, the signal photons can be efficiently frequency up-converted, while background noise photons carrying different temporal modes are converted with much lower probabilities.

## Data Availability

Data underlying the results presented in this paper are not publicly available at this time but may be obtained from the corresponding author on reasonable request.
